# Copper-catalyzed reactions of β-alkoxy/phenoxy enones with dimethyl diazomalonate

**DOI:** 10.55730/1300-0527.3519

**Published:** 2022-01-04

**Authors:** Füsun Şeyma GÜNGÖR

**Affiliations:** Department of Chemistry, Faculty of Science and Letters, İstanbul Technical University, İstanbul, Turkey

**Keywords:** Carbene, metal-carbenoid, carbonyl ylide, 2,3-dihydrofuran, cycloaddition

## Abstract

2,3-dihydrofurans were synthesized from carbonyl-ylides via 1,5-electrocyclization reactions with high yields. Dimethyl diazomalonate was reacted with several β-alkoxy and/or β-phenoxy α,β-unsaturated compounds in the presence of Cu(acac)_2_ as a catalyst. From the reaction of β-methoxy enone with diazo compound, dioxole, and Cα-H insertion products were also obtained as side products along with 2,3-dihydrofuran derivative. When the unsaturated compound has an ester and a ketone group, only one dihydrofuran derivative was formed, which occurred by the 1,5-ring closure of keto-carbonyl ylide. Dihydrofuran derivative from the formation of ester carbonyl ylide in the reactions was not obtained.

## 1. Introduction

Substituted dihydrofurans are intermediates for the synthesis of a wide variety of compounds such as furan, cyclopropyl aldehyde, γ-hydroxy aldehyde, γ-hydroxy ketone, γ-lactone, and hydroxy amino acid [1–2]. 2,3-Dihydrofuran structures are also found in natural compounds [3]. A literature survey reveals that many different strategies have been described for the synthesis of 2,3-dihydrofurans [4–12].

**Figure 1:**
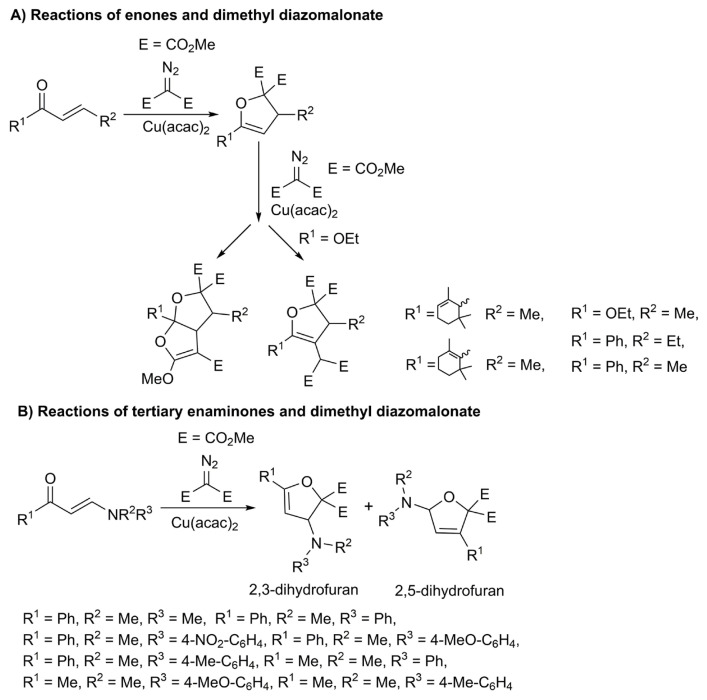
Synthesis of 2,3-dihydrofurans.

One of the existing strategies for the synthesis of dihydrofuran in the literature is the reaction of α,β-unsaturated enone compounds and diazo compounds in a catalytic medium. Spencer et. al. reported that the CuSO_4_-catalyzed reactions of β-methoxy α,β-unsaturated ketone with ethyl diazoacetate gave furan compounds over 2,3-dihydrofurans via methanol elimination [13–14]. After this pioneering study, the reactions of enones with diazo compounds have been realized in the presence of several catalysts [15–17]. 2,3-Dihydrofurans were obtained in good yields under suitable reaction conditions in these studies (Figure 1A). In another report, significant amounts of 2,3-dihydrofurans along with 2,5-dihydrofurans were obtained by the Cu(acac)_2_-catalyzed reactions of tertiary enaminones and dimethyl diazomalonate (Figure 1B) [18–20]. Accordingly, it is worth investigating the effects of OR instead of NR_2_ groups on the α,β-unsaturated carbonyl compounds for the formation of 2,3-dihydrofurans. From the sole example of the reactions of β-alkoxy α,β-unsaturated carbonyl compounds and ethyl diazoacetate, cyclopropane derivatives were obtained [21]. However, the formation of cyclopropane products is expected from these reactions due to the *s*-trans conformation of β-alkoxy α,β-unsaturated substrates. In another study, Son and Fu demonstrated copper-catalyzed asymmetric [4+1] cycloaddition reactions of enones with diazo compounds to obtain 2,3-dihydrofurans in good yield with high stereoselectivity [22]. Moreover, they also reported one example of the synthetic 2,3-dihydrofuran from the copper-catalyzed reactions of β-alkoxy α,β-unsaturated ketone and aryl diazoacetate. From this perspective, we aimed to investigate the copper-catalyzed reactions of β-alkoxy/phenoxy α,β-unsaturated compounds and dimethyl diazomalonate in this study.

## 2. Experimental section

### 2.1. General

All solvents and reagents were supplied commercially as reagent grade. Compounds **1b** and **1c** were purchased by Sigma-Aldrich. Dimethyl diazomalonate was synthesized according to the literature [23]. All reactions of dimethyl diazomalonate were carried out under a nitrogen atmosphere. NMR spectra were recorded on Bruker AC (^1^H NMR: 250 MHz, ^13^C NMR: 60 MHz) and Agilent VNMRS (^1^H NMR: 500 MHz, ^13^C NMR: 125 MHz). Chemical shifts (*δ*) are reported in ppm with respect to the internal standard tetramethylsilane (TMS). Splitting patterns were described as follows: s (singlet), d (doublet), t (triplet), q (quartet), p (quintet), m (multiplet), and bs (broad singlet). GC-MS analyses were performed on a Thermo Finnigan trace DSQ instrument equipped with a flame ionization detector. A 5% Phenyl polyphenylene-siloxane capillary column (TR-5MS) was used with helium as the carrier gas. The temperature program is as follows: Start 100 °C 5 min isothermal, ramp 20 °C, final 290 °C, and then 10 min isothermal. Retention times (*t*_R_) are reported in a minute. Melting points were recorded on the Buchi Melting Points B-540 apparatus. HR-MS: Agilent 6230-B TOF LC/MS in m/z.

### 2.2. Synthesis of β-Alkoxy/Phenoxy α,β-Enones

#### Preparation of β-Keto Aldehyde Sodium Enolate (Step 1)

To a solution of sodium hydride (80%, 0.8 mol) in diethyl ether (400 mL) was added methanol (0.8 mol) at reflux temperature. After addition, the mixture was refluxed for 10 min and cooled at 0 °C. The mixture of methyl ketone (0.8 mol) and methyl formate (0.84 mol) was added to the mixture at 5–10 °C in 40 min. After the addition of diethyl ether (200 mL), the mixture was stirred overnight. Then, the mixture was filtered, and the precipitate was washed with 200 mL of diethyl ether. β-Keto aldehyde sodium enolate was dried in vacuo.

#### Preparation of β-Acylethenyl Chloride (Step 2)

β-Keto aldehyde sodium enolate (1 equiv.) was dissolved in cold H_2_O and extracted with CH_2_Cl_2_. The aqueous layer added 2 N acetic acid (1 equiv.), and the mixture was extracted with CH_2_Cl_2_. The organic layer was washed with H_2_O and brine and dried over MgSO_4_. The solvent was removed in vacuo. The residue (β-Ketoaldehyde) (100 mmol) was dissolved in benzene (100 mL) and thionyl chloride (110 mmol) was added to this solution. The mixture was refluxed until HCl no longer evolved. The solvent was removed under reduced pressure, and the residue was distilled in vacuo (yield 65%).

#### Preparation of β-Phenoxy α,β-Enones (Step 3)

A solution of β-acylethenyl chloride (18 mmol) in 50 mL of benzene and phenolic compound (18 mmol) in 50 mL of 10% NaOH solution were mixed. After the addition of tetrabutylammonium bromide (1.6 mmol) to the solution, the reaction mixture was stirred at 60 °C for 24 h. The benzene layer was removed and the aqueous layer was extracted with benzene (10 mL). The organic layer was combined, washed with H_2_O, and dried over MgSO_4_. Benzene was removed in vacuo and the residue was purified by recrystallization from hexane.

#### (*E*)-1-Phenyl-3-(*p*-tolyloxy)prop-2-ene-1-one (**1a**)

Acetophenone was used as a methyl ketone in Step 1 and toluidine was used as a phenolic compound in Step 3. The product was synthesized according to Step 1, Step 2, and Step 3, respectively. Obtained as yellow solid, m.p.: 85–88 °C; ^1^H NMR (250 MHz, CDCl_3_): *δ* 7.96 (d, *J* = 11.8 Hz, 1H, C=C*H*-OC_6_H_4_-*p*-CH_3_), 7.90 (d, *J* = 7.6 Hz, Ar-*H*, 2H), 7.54–7.50 (m, Ar-*H*, 1H), 7.45 (distorted t, *J* = 7.7–6.9 Hz, Ar-*H*, 2H), 7.17 (d, *J* = 8.6 Hz, Ar-*H*, 2H), 7.00 (d, *J* = 8.4 Hz, Ar-*H*, 2H), 6.68 (d, *J* = 11.7 Hz, *H*C = CH-OC_6_H_4_-*p*-CH_3_, 1H), 2.34 (s, C = CH-OC_6_H_4_-*p*-C*H_3_*,3H); ^13^C NMR (60 MHz, CDCl_3_): *δ* 190.4 (*C* = O), 160.7 (C = *C*-O-), 154.0, 138.4, 134.8, 132.5, 130.4, 128.5, 128.1, 117.8, 106.4, 20.7 (*C*H_3_); *t*_R_ = 8.18; EI-MS (m/z): 178 (M^+^, 94), 147 (100), 118 (38), 91 (61), 77 (81), 51 (47); HR-MS: calcd for C_16_H_16_O_2_ [M+H]^+^ 239.1072, found 239.1081.

#### Ethyl 2-(ethoxymethylene)-3-oxobutanoate (**1d**) [24]

Ethyl acetoacetate (3 mol), triethyl orthoformate (3.6 mol), and glacial acetic acid (9 g) were placed in 2 L of a three-necked round-bottom flask, which is equipped with a thermometer and distillation set-up. The system was heated and alcohol was distilled about at 125 °C. The reaction took place at about 4 h. Product was purified by vacuum distillation (at 152–158 °C/22 mmHg, yield 50%). The product obtained as brown oil; EI-MS (m/z): 186 (M^+^, 5), 171 (94), 143 (51), 115(100), 97 (49), 71 (42), 53 (7); Isomer 1: *t*_R_ = 8.87; ^1^H NMR (250 MHz, CDCl_3_): *δ* 7.48 (s, =C*H*OCH_2_CH_3_, 1H), 4.09–3.97 (m, OC*H_2_*CH_3_, 4H), 2.19 (s, C*H_3_*CO, 3H), 1.23–1.07 (m, OCH_2_C*H_3_*, 6H); Isomer 2: *t*_R_ = 9.06; ^1^H NMR (250 MHz, CDCl_3_): *δ* 7.47 (s, =C*H*OCH_2_CH_3_, 1H), 4.09-3.97 (m, OC*H_2_*CH_3_, 4H), 2.12 (s, C*H_3_*CO, 3H), 1.23–1.07 (m, OCH_2_C*H_3_*, 6H).

### 2.3. General procedure for the reaction of β-Alkoxy/Phenoxy α,β-Enones with Dimethyl Diazomalonate

Cu(acac)_2_ (0.01 mmol, 0.007 equiv.) and a solution of β-alkoxy/phenoxy enone (2.1 mmol, 1.5 equiv.) in benzene (10 mL) were heated under reflux. A solution of dimethyl diazomalonate (1.4 mmol, 1 equiv.) in benzene (1.5 mL) was added to this solution over 2.5 h under a nitrogen atmosphere. When the IR spectrum of the reaction mixture indicated the absence of a characteristic diazo band at 2130 cm^−1^, the mixture was filtered, concentrated, and purified by silica column chromatography.

#### Dimethyl 5-phenyl-3-(p-tolyloxy)furan-2,2(3H)-dicarboxylate (**3a**)

Isolated by silica column chromatography using hexane: ethyl acetate (4:1) as an eluent. Obtained yellow oily compound (yield 76 %); ^1^H NMR (250 MHz, CDCl_3_): *δ* 7.70 (dd, *J* = 8.8/8.0 Hz, Ar-*H*, 2H), 7.51–7.32 (m, Ar-*H*, 3H), 7.04 (d, *J* = 8.5 Hz, Ar-*H*, 2H), 6.92 (d, *J* = 8.6 Hz, Ar-*H*, 2H), 4.55 (bs, C*H*-O, 1H), 4.17 (bs, C=C*H*, 1H), 3.85 (s, CO_2_C*H*_3_, 3H), 3.49 (s, CO_2_C*H*_3_, 3H), 2.25 (s, C*H*_3_, 3H); ^13^C NMR (60 MHz, CDCl_3_): *δ* 168.0 (*C*O_2_CH_3_), 166.4 (*C*O_2_CH_3_), 156.8, 145.7 (O-*C*(Ph) = C), 132.7, 131.6, 130.4, 129.6, 128.4, 126.8, 117.6, 102.5 (O-C(Ph)=*C*), 93.4 (*C*(CO_2_CH_3_)_2_), 82.9 (*C*-OPh), 52.7 (CO_2_*C*H_3_), 52.2 (CO_2_*C*H_3_), 20.8 (*C*H_3_); *t*_R_ = 14.0; EIMS (m/z): 368 (M^+^, 2), 281 (11), 230 (84), 202 (100), 105 (51), 77 (41), 59 (8); HR-MS: calcd for C_21_H_21_O_6_ [M+H]^+^ 369.1333, found 336.1320.

#### Dimethyl 3-methoxy-5-methylfuran-2,2(3H)-dicarboxylate (**3c**)

Isolated by silica column chromatography using hexane: ethyl acetate gradually from beginning 4:0.4 as an eluate. **3c** was obtained with compound **4** as a mixture of pale-yellow oil (**3c**:**4**, 6:1 from ^1^H NMR). ^1^H NMR (500 MHz, CDCl_3_): *δ* 5.14 (quintet, *J* = 1.3 Hz, C*H*OCH_3_, 1H), 4.91 (q, *J* = 1.3 Hz, =C*H*, 1H), 3.84 (s, CO_2_C*H*_3_, 3H), 3.83 (s, CO_2_C*H*_3_, 3H), 3.36 (s, OC*H*_3_, 3H), 1.95 (dd, *J* = 1.8/1.3 Hz, C*H*_3_, 3H); ^13^C NMR (125 MHz, CDCl_3_): *δ* 167.3 (*C*O_2_CH_3_), 165.5 (*C*O_2_CH_3_), 159.1 (O-*C*=C), 96.6 (O-C=*C*), 91.7 (*C*(CO_2_CH_3_)_2_), 87.3 (*C*H-OCH_3_), 57.7 (CH-O*C*H_3_), 53.0 (CO_2_*C*H_3_), 52.9 (CO_2_*C*H_3_), 13.7 (*C*H_3_); *t*_R_ = 8.34; EI-MS (m/z): 230 (M^+^, 11) 198 (23), 170 (69), 155 (65), 139 (41), 109 (100), 83 (27), 69 (62), 59 (45).

#### 4-Ethyl 2,2-dimethyl 3-ethoxy-5-methylfuran-2,2,4(3H-tricarboxylate) (**3d**)

Isolated by silica preparative thin layer chromatography using hexane: ethyl acetate (4:1) as an eluate (yield 84 %); ^1^H NMR (250 MHz, CDCl_3_): *δ* 5.28 (s, C*H*-OCH_2_CH_3_, 1H), 4.18–4.03 (m, CO_2_C*H*_2_CH_3_, 2H), 3.76 (s, CO_2_C*H*_3_, 6H), 3.68–3.50 (m, OC*H*_2_CH_3_, 2H), 2.23 (s, C*H*_3_, 3H), 1.20 (t, *J* = 7.2 Hz, OCH_2_C*H*_3_, 3H), 1.04 (t, *J* = 7.0 Hz, OCH_2_C*H*_3_, 3H); ^13^C NMR (60 MHz, CDCl_3_): *δ* 168.8 (*C*O_2_CH_3_), 165.2 (*C*O_2_CH_3_), 163.6 (*C*O_2_CH_2_CH_3_), 163.3 (O-*C*=C), 104.6 (*C*=C-O), 90.9 (*C*(CO_2_CH_3_)_2_), 83.9 (*C*H-OCH_2_CH_3_), 67.3 (CO_2_*C*H_2_CH_3_), 58.9 (O*C*H_2_CH_3_), 52.6 (CO_2_*C*H_3_), 52.0 (CO_2_*C*H_3_), 14.4 (OCH_2_*C*H_3_), 13.3 (*C*H_3_); *t*_R_ = 11.8; EI-MS (m/z): 316 (M^+^, 1), 301 (4), 271 (51), 240 (71), 227 (100), 212 (60), 183 (81), 167 (61), 123 (21), 59 (36); HR-MS: calcd for C_14_H_21_O_8_ [M+H]^+^ 317.1231, found 317.1257.

#### Methyl (E)-5-methoxy-2-(2-methoxyvinyl)-2-methyl-1,3-dioxole-4-carboxylate (**4**)

Isolated by silica column chromatography using hexane: ethyl acetate gradually from beginning 4:0.4 as an eluate. Compound **4** was obtained with **3c** as a mixture of pale-yellow oil (**3c**:**4**, 6:1 from ^1^H NMR); ^1^H NMR (500 MHz, CDCl_3_): *δ* 6.25 (d, *J* = 5.6 Hz, C*H*=OCH_3_, 1H), 5.95 (d, *J* = 5.6 Hz, C=C*H*-C, 1H), 3.85 (s, 3H, OC*H*_3_), 3.83 (s, CO_2_C*H*_3_, 3H), 3.81 (s, OC*H*_3_, 3H), 1.61 (s, *C*H_3_, 3H); ^13^C NMR (125 MHz, CDCl_3_): *δ* 169.4 (C=*C*-OCH_3_), 164.8 (*C*O_2_CH_3_), 133.4 (*C*H(OCH_3_)=CH), 128.9 (*C*-CO_2_CH_3_), 116.2 (CH(OCH_3_)=*C*H), 91.4 (*C*-CH_3_), 53.5 (O*C*H_3_), 53.3 (CO_2_*C*H_3_), 50.4 (O*C*H_3_), 25.7 (*C*H_3_); *t*_R_ = 8.12; EI-MS (m/z): 230 (M^+^, 1) 215 (10), 199 (20), 171 (100), 155 (32), 139 (50), 111 (77), 109 (67), 83 (61), 59 (59).

#### Dimethyl (E)-2-(1-methoxy-3-oxobut-1-ene-2-yl)malonate (**5**)

Isolated by silica column chromatography using hexane: ethyl acetate gradually from beginning 4:0.6 as an eluent (yield 10 %); ^1^H NMR (500 MHz, CDCl_3_): *δ* 7.40 (s, C=C*H*, 1H), 3.93 (s, OC*H*_3_, 3H), 3.73 (s, CO_2_C*H*_3_, 6H), 4.69 (s, C*H*, 1H), 2.27 (s, C*H*_3_CO, 3H); ^13^C NMR (125 MHz, CDCl_3_): *δ* 194.3 (*C*=O), 168.5 (*C*O_2_CH_3_), 162.6 (C=*C*H-OCH_3_), 116.3(*C*=CH-OCH_3_), 62.4 (O*C*H_3_), 52.6 (CO_2_*C*H_3_), 46.9 (*C*H), 24.9 (*C*H_3_CO); *t*_R_ = 10.1; EI-MS (m/z): 229 (17), 187 (74), 159 (66), 115 (100), 69 (86), 59 (82); HR-MS: calcd for C_10_H_15_O_6_ [M+H]^+^ 231.0891, found 231.0915.

## 3. Result and discussion

**Scheme 1:**
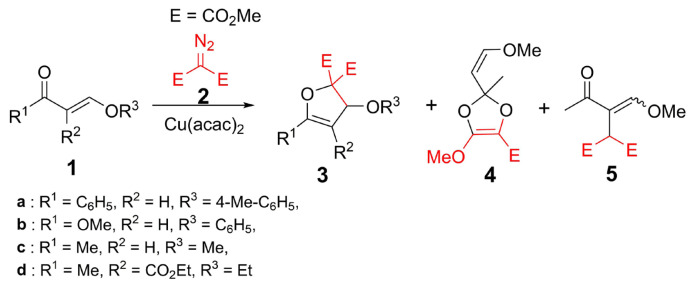
Copper-catalyzed reactions of β-alkoxy/phenoxy enones and dimethyl diazomalonate.

From our previous reports, dihydrofurans and dihydroxepines were obtained from the reactions of α,β-unsaturated carbonyls (such as enones and enaminones) and dimethyl diazomalonate in the presence of Cu(acac)_2_ catalyst [10–15]. Accordingly, the Cu(acac)_2_-catalyzed reactions of β-alkoxy/phenoxy enones and dimethyl diazomalonate were investigated in this study (Scheme 1). Initially, we performed model reactions with (*E*)-1-phenyl-3-(*p*-tolyloxy)prop-2-ene-1-one (**1a**) and dimethyl diazomalonate and different copper catalysts (Table 1). Accordingly, Cu(acac)_2_ displayed the best catalytic performance for the intended reactions (Table 1).

After determining the catalyst, the reactions of some β-alkoxy/phenoxy enones and dimethyl diazomalonate in the presence of Cu(acac)_2_ (Scheme 1) were performed and the results are summarized in Table 2. The groups of R^1^ and R^2^ in the substrate structure were chosen based on the presence of the carbonyl group as a ketone, ester, or both ketone and ester function in the same structure. Thus, it was aimed to determine the reactivity of various carbonyl groups in the structure against metal-carbenoid.

In the Cu(acac)_2_-catalyzed reaction of (*E*)-1-phenyl-3-(*p*-tolyloxy)prop-2-ene-1-one (**1a**) and dimethyl diazomalonate, dimethyl 5-phenyl-3-(*p*-tolyloxy)furan-2,2(3*H*)-dicarboxylate (**3a**) was obtained as the sole product (Table 2). On the contrary, substrate **1b** (R^1^ = OMe) did not give any product with the diazo compound. However, a dimerization reaction of carbene occurred and only carbene dimers were obtained.

The reaction of the dimethyl diazomalonate with β-methoxy enone (R^3^ = Me, **1c**), yielded a 2,3-dihydrofuran derivative along with dioxole (**4**) and insertion (**5**) products. The widespread product distribution is observed in this reaction since (*E*)-4-methoxy-but-3-ene-2-one (**1c**) is less hindered than other reactants (**1a**, **1b**, **1d**) and the reaction could not proceed chemoselectively. Carbonyl ylide intermediate of (*E*)-4-methoxybut-3-ene-2-one (**1c**) and copper-carbenoid gave a 1,5-electrocyclization reaction and formed 2,3-dihydrofuran (**3c**) as a major product.

**Scheme 2:**
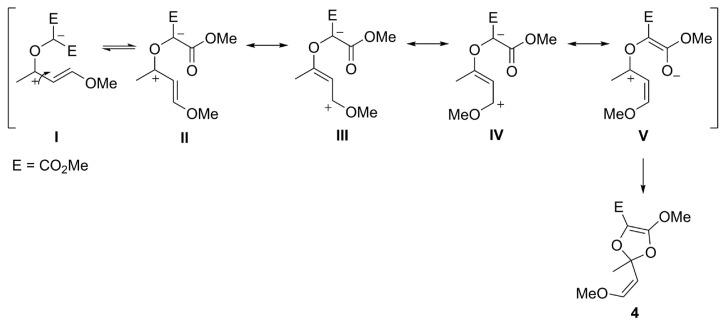
Possible route for the formation of dioxole (**4**).

Concomitantly, dioxole (**4**) derivative as another cyclization product also occurred from the carbonyl ylide intermediate (Scheme 2). Intermediate **III** can be formed when rotation around a single bond occurs in intermediate **I** and the negative charge is distributed from the carbene carbon to the ester group. With the cyclization of intermediate **V**, the dioxole product is formed. Despite the chromatographic purification attempts, dioxole (**4**) was obtained as a mixture with **3c.**

**Scheme 3:**
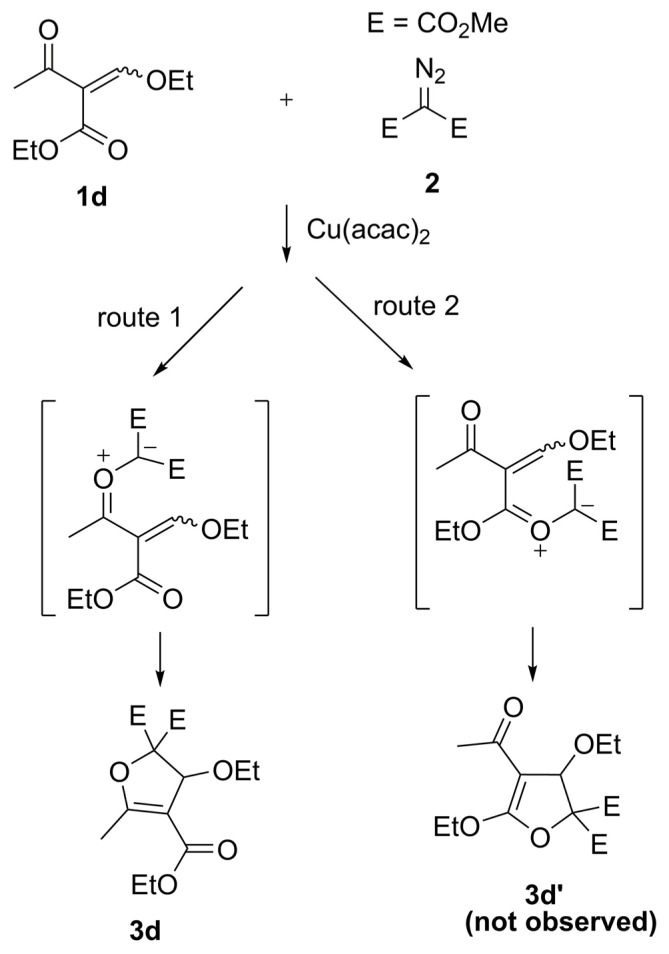
Formation of product **3d.**

In the same reaction, the attack of copper-carbenoid to Cα-H bond in (*E*)-4-methoxy-but-3-ene-2-one (**1c**) formed **5** as a minor product. Here it should be noted that no insertion product was observed in other reactions. However, 2,3-dihydrofuran (**3d**) was obtained as the sole product with a high yield in the copper-catalyzed reaction of ethyl 2-(ethoxymethylene)-3-oxobutanoate (**1d**) and dimethyl diazomalonate. Compound **1d** has an ester and a ketone group in its structure, and **1d** has been synthesized as two isomers. Only one of the carbonyl groups reacted with carbene to form the dihydrofuran compound (**3d**) (route 1, Scheme 3). The formation of this dihydrofuran can only occur by the 1,5-ring closure of the keto-carbonyl ylide. On the other hand, no 1,5-cyclization product (**3d′**) was observed from the ester-carbonyl ylide (route 2, Scheme 3). This result agrees with our previous studies [16–17, 19–20].

## 4. Conclusion

Cu(acac)_2_-catalyzed reactions of β-alkoxy/phenoxy α,β-unsaturated carbonyls with dimethyl diazomalonate gave 2,3-dihydrofurans. The carbonyl compounds used in these reactions are electron-rich conjugated systems due to β-alkoxy/phenoxy functional groups. The electron-rich nature of these carbonyl oxygen atoms facilitated the electrophilic attack of copper-carbenoid. Thus, 2,3-dihydrofuran products were mainly formed in good yields over carbonyl-ylides. In these reactions, a dioxole derivative and Cα-H insertion product were also observed only in one reaction as side products. When the β-alkoxy enone has both an ester and ketone groups, the dihydrofuran derivative was formed only from the keto-ylide via 1,5-electrocyclization.

## Figures and Tables

**Table 1:** Catalyst optimizations for the reactions of (*E*)-1-phenyl-3-(*p*-tolyloxy)prop-2-ene-1-one and dimethyl diazomalonate.^a^

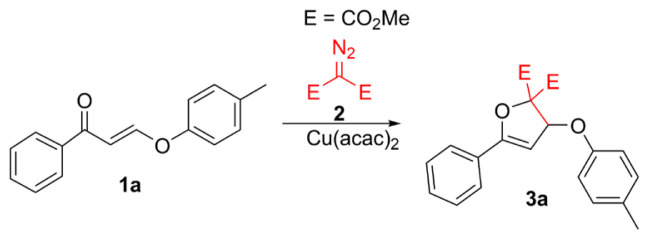			
Entry	Catalyst	Time (h)	Isolated yield (3a) %
1	CuCl_2_	24	30
2	Cu(acac)_2_	18	76
3	Cu(hfacac)_2_	24	9
4^b^	Cu(OTf)_2_	20	-

a The reactions were carried out with **1a** (2.1 mmol), diazo compound (1.4 mmol), catalyst (0.01 mmol), and dry benzene (10 mL) at 80 °C under a nitrogen atmosphere.

b Only carbene dimers were obtained.

**Table 2:** Reactions of β-alkoxy/phenoxy enones and dimethyl diazomalonate^a^.

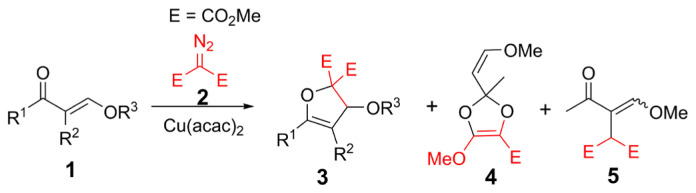						
1	R^1^	R^2^	R^3^	3 (yield %)^b^	4 (yield %)^b^	5 (yield %)^b^
**1a**	C_6_H_5_	H	4-Me-C_6_H_5_	**3a** (76)^b^	-	-
**1b**	OMe	H	C_6_H_5_	-	-	-
**1c**	Me	H	Me	**3c**:**4** (40)^c^ (6:1)^d^	**3c**:**4** (40)^c^ (6:1)^d^	**5** (10)^b^
**1d**	Me	CO_2_Et	Et	**3d** (84)^b^	-	-

a The reactions were carried out with **1a** (2.1 mmol), diazo compound (1.4 mmol), catalyst (0.01 mmol), and dry benzene (10 mL) at 80 °C under a nitrogen atmosphere.

b Isolated yield from the reaction.

c The amount of product **3c** in the crude mixture was determined by GC.

d Relative product ratios were determined by ^1^H NMR.

## References

[1] Gaudry R The synthesis of amino acids from 2,3-dihydrofuran DL-ornithine, DL-proline, and DL-α-amino-δ-hydroxyvaleric acid Canadian Journal of Chemistry 1951 29 7 544 551 10.1139/v51-063 14848757

[2] Wilson CL Reactions of furan compounds. VII. Thermal interconversion of 2,3-dihydrofuran and cyclopropane aldehyde Journal of American Chemical Society 1947 69 12 3002 3004 10.1021/ja01204a020

[3] Von Dreele RB Pettit GR Ode RH Perdue RE White JD Crystal and molecular structure of unusual spiro dihydrofuran diterpene nepetaefolin Journal of American Chemical Society 1975 97 21 6236 6240 10.1021/ja00854a049 1176729

[4] Kilroy TG O’Sullivan TP Guiry PJ Synthesis of dihydrofurans substituted in the 2-position European Journal of Organic Chemistry 2005 23 4929 4949 10.1002/ejoc.200500489

[5] Wang Q-F Hou H Hui L Yan C-G Diastereoselective synthesis of trans-2,3-dihydrofurans with pyridinium ylide assisted tandem reaction Journal of Organic Chemistry 2009 74 19 7403 7406 10.1021/jo901379h 19739607

[6] Yang Z Fan M Liu W Liang Y A Novel facile synthesis route to highly substituted 2,3-dihydrofurans via ammonium ylides Synthesis 2005 13 2188 2192 10.1055/s-2005-869956

[7] Zhao L-B Guan Z-H Han Y Xie Y-X He S Copper-catalyzed [4+1] cycloadditions of α,β-acetylenic ketones with diazoacetates to form trisubstituted furans Journal of Organic Chemistry 2007 72 26 10276 10278 10.1021/jo7019465 18041850

[8] Yılmaz M Biçer E Pekel AT Manganese (III) acetate mediated free radical cyclization of 1,3-dicarbonyl compounds with sterically hindered olefins Turkish Journal of Chemistry 2005 29 579 587 https://journals.tubitak.gov.tr/chem/vol29/iss6/1

[9] Çalışkan R Ali MF Şahin E Watson WH Balcı M Unusual manganese (III)-mediated oxidative free radical additions of 1,3-dicarbonyl compounds to benzonorbornadiene and 7-heterobenzonorbornadienes: Mechanistic studies Journal of Organic Chemistry 2007 72 9 3353 3359 10.1021/jo0625711 17385919

[10] Demir AS Emrullahoğlu M Manganese (III) acetate: A versatile reagent in organic synthesis Current Organic Synthesis 2007 4 321 350 10.2174/157017907781369289

[11] Südemen MB Zengin M Genç H Balcı M Reactions of heptatriene derivatives with 1,3-diketones in the presence of Mn(OAc)_3_ Turkish Journal of Chemistry 2011 35 1 11 10.3906/kim-1011-776

[12] Deliomeroglu MK Dengiz Ç Çalışkan R Balcı M Mn(OAc)_3_-promoted sulfur-directed addition of an active methylene compound to alkenes Tetrahedron 2012 68 5838 5844 10.1016/j.tet.2012.05.003

[13] Storm DL Spencer TA Furan synthesis by 1,4-addition of carboethoxycarbene to α-methoxymethylene ketones Tetrahedron Letters 1967 8 20 1865 1867 10.1016/S0040-4039(00)90743-3

[14] Spencer TA Villarica RM Storm DL Weaver TD Friary RJ Total synthesis of racemic methyl vinhaticoate Journal of American Chemical Society 1967 89 21 5497 5499 10.1021/ja00997a060

[15] Anac O Daut A Reactions of α,β-enones with diazo compounds. Part 2: Synthesis of dihydrofuran derivatives Liebigs Annalen/Recuil 1997 6 1249 1254 10.1002/jlac.199719970630

[16] Anac O Gungor FS Kahveci C Cansever MS Reactions of α,β-enones with diazo compounds. Part 4: Reaction pathways from (Z)- and (E)- α,β-enones with dimethyl diazomalonates Helvetica Chimica Acta 2004 87 2 408 415 10.1002/hlca.200490039

[17] Anac O Gungor FS Merey G Synthesis of highly functionalized γ-lactones via 1,5-electrocyclic ring closure Helvetica Chimica Acta 2006 89 6 1231 1240 10.1002/hlca.200690120

[18] Gungor FS Anac O Sezer O Synthesis of naphthalenone, dihydroquinoline, and dihydrofuran derivatives Helvetica Chimica Acta 2011 94 6 1115 1129 10.1002/hlca.201000386

[19] Gungor FS Hancioglu N Anac O Reactions of enaminones with diazo carbonyl compounds Helvetica Chimica Acta 2013 96 3 488 493 10.1002/hlca.201200233

[20] Gungor FS Anac O Sezer O Observations on the copper(II) catalyzed reactions of enaminones and dimethyl diazomalonate Tetrahedron Letters 2007 48 28 4883 4886 10.1016/j.tetlet.2007.05.063

[21] Rotzoll S Appel B Langer P Synthesis of 2,3-benzoxepins by sequential cyclopropanation/ring-enlargement reactions of benzopyrylium triflates with diazoesters Tetrahedron Letters 2005 46 23 4057 4059 10.1016/j.tetlet.2005.04.009

[22] Son S Fu GC Copper-catalyzed asymmetric [4+1] cycloadditions of enones with diazo compounds to form dihydrofurans Journal of American Chemical Society 2007 129 5 1046 1047 10.1021/ja068344y PMC2569997 17263382

[23] Wulfman DS McGibboney BG Steffen EK Thinh NV Mcdaniel RS Metal salt catalyzed carbenoids-VX: The synthetic and structural aspects of copper salt catalyzed additions of bis-methoxycarbonyl carbene to olefins Tetrahedron 1976 32 11 1257 1265 10.1016/0040-4020(76)80080-4

[24] Nyack LN Tarsio PJ Point S Blohm H Process for preparation of oxy alkylidene compounds US Patent 1958 2824121 https://worldwide.espacenet.com/patent/search/family/023853689/publication/US2824121A?q=pn%3DUS2824121A

